# Treatment of direct carotid-cavernous fistula with Willis covered stent with midterm follow-up

**DOI:** 10.1186/s41016-021-00256-y

**Published:** 2021-09-13

**Authors:** Qinglin Liu, Changjing Qi, Yunyan Wang, Wandong Su, Gang Li, Donghai Wang

**Affiliations:** 1 Department of Neurosurgery, Beijing Tiantan Hospital, Capital Medical Universitys, 119#, Nansihua Xi Road, Fengtai District, Beijing, 100050 China; 2 Nursing Department of Qilu Hospital, Shandong University, 107# Wenhua Xi Road, Jinan, 250012 Shandong province China; 3 Neurosurgery Department of Qilu Hospital, Shandong University, 107# Wenhua Xi Road, Jinan, 250012 Shandong province China

**Keywords:** Fistula, Endovascular procedures, Stents, Endoleak

## Abstract

**Background:**

Willis covered stent is the first stent designed exclusively for intracranial vasculature, and its application in carotid-cavernous fistula is limited. The aim is to evaluate the feasibility and efficacy of this device in treating direct carotid-cavernous fistula.

**Methods:**

Ten consecutive patients with direct carotid-cavernous fistula were treated in our institution with Willis covered stents from September 2013 to December 2015. The characteristics of these patients and the immediate and follow-up results were retrospectively reviewed.

**Results:**

Of the 10 patients, 8 were treated for the first time, and 2 had been treated elsewhere. Willis covered stents were successfully released in 9 patients. Abnormal arteriovenous shunt disappeared in 6 cases immediately after stent deployment and endoleak occurred in 3 cases. Endoleak disappeared at 6-month angiography follow-up in one case and was sealed with coils through a pre-set microcatheter in another case. Parent artery was sacrificed as endoleak remained despite repeated balloon dilation and a second stent deployment in the third case. All patients got clinical follow-ups for at least 24 months and 7 patients received angiographic follow-up. Symptoms were relieved gradually in all cases except for slight oculomotor paralysis and visual acuity in one case, respectively. In-stent stenosis was found in 1 case, and no recurrence was observed.

**Conclusions:**

Willis covered stent is feasible for direct carotid-cavernous fistula.

## Background

Direct carotid-cavernous fistula (CCF) is featured by a direct shunt between the internal carotid artery (ICA) and the cavernous sinus, which mainly results from head traumas that damaged the cavernous ICA. Spontaneous direct CCF may also occur after rupture of a cavernous ICA aneurysm. The typical clinical manifestation of a direct CCF is a triad consisted of pulsating exophthalmos, chemosis, and visual loss. Stealing of the blood may result in hypoperfusion of the ipsilateral hemisphere and the drainage pattern determines the hazardous consequences of the disease. Cortical superficial vein drainage may cause lethal intracranial hemorrhage.

The optimal goal for treating CCFs is to occlude the abnormal shunt and preserve ICA patency. In 1973, Parkinson first reported surgical repair of direct CCFs with parent artery preservation, but the technical difficulty and significant morbidity have precluded its widespread use [1]. In 1974, Serbinenko reported his experience with detachable balloons for treating CCFs [2]. This technique results in closure of the fistula with ICA preservation in up to 80% of cases and has been the first-line therapy for direct CCFs [3]. Easy delivery and low cost are the main advantages of this technique. However, it also has some technical problems such as early detachment/deflation of the balloon or occasional rupture of the balloon stabbed by the bone fragments [4]. Detachable coils were used in 1992 by Guglielmi with the advantages of thrombogenicity, controllable deposit, radiopacity, and biocompatibility [5]. Dense packing of the cavernous sinus might cause cranial nerve paralysis. Furthermore, the coils may protrude into the ICA and cause embolic events. Liquid embolic agents including n-butyl cyanoacrylate and Onyx have also been used solely or in combination with coils in treating direct CCFs with favorable results [6, 7]. The potential of the embolic agent refluxing into the ICA or draining veins is the main disadvantage of this approach [8].

Recently, covered stents for treating CCFs have been reported [4, 9, 10]. But all these covered stents were not initially for neurovascular use. As the first device designed exclusively for intracranial vascular defect, the Willis covered stent (MicroPort, Shanghai, China) was approved by the Chinese Food and Drug Administration in 2013. It is a balloon-expandable endoprosthesis composed of a bare stent and an expandable polytetrafluoroethylene membrane. Recently, it has been used for aneurysms [11] and arterial dissections [12] with satisfying results. However, its application for CCF is still limited [9]. In this study, we will explore the feasibility and safety of this stent in treating direct CCF.

## Methods

### Patient cohort

From September 2013 to December 2015, 10 patients with traumatic direct CCF received Willis covered stent implantation in our institution. This study was approved by the Ethics Committee of Qilu Hospital (No. KYLL-2018-138), and informed consent was obtained from all the patients before operation. The angiographic data was reviewed by 3 senior neuro-radiologists for screening the candidates for Willis covered stent treatment. The major considerations were the tortuosity of the parent artery, the presence of vital perforators, and the contradictions for antiplatelet therapy. Details of alternative treatment strategies such as embolization with coils, ethylene vinyl alcohol copolymer (Onyx, Covidien, Minneapolis, MN), and balloon were informed to the patients and their relatives, and the patients made the final treatment decision.

### Endovascular procedure

All patients received a diagnostic 6-vessel digital subtraction angiography (DSA). Angiographic evaluation included ipsilateral vertebral and contralateral carotid angiographies with ipsilateral carotid compression. Aspirin (Bayer Pharma AG., Leverkusen, Germany) and clopidogrel (Bristol-Myers Squibb/Sanofi Pharmaceuticals, Bridgewater, NJ, 300 mg, respectively) were prescribed 2 h before the operation. All the procedures were performed under general anesthesia using the Siemens Artis Zee floor system (Siemens, Germany). Heparinization was monitored with an activated clotting time of 250–300 s.

An 8F guiding catheter (Boston Scientific Co., USA) was placed into the internal carotid artery. Through this stiff guiding catheter, a 5F Navien (EV3, Irvine, CA, USA) catheter, an XT-27 microcatheter (Stryker, Kalamazoo, MI, USA), and a 0.014-inch Synchro microwire (Stryker, West Valley City, Utah, USA) were delivered in co-axis. The distal tip of the 5F Navien catheter should be delivered across the fistula and to the ICA bifurcation. Then, the Synchro microwire was exchanged for a 300 cm exchanging microwire (Stryker, West Valley City, Utah, USA), and the XT-27 microcatheter was withdrawn. Over the exchanging microwire, the Willis covered stent was delivered to cover the fistula. Then, the 5F Navien catheter was withdrawn gradually to the proximal of the fistula carefully while keeping the stent stable. After confirming the position of the stent with angiography from the Navien catheter, the balloon was dilated to release the stent. Subsequent angiography was obligated to evaluate the result. Repeated balloon inflation, another stent deployment, or parent artery occlusion would be considered for unexpected endoleak.

For patient 4, a “pre-set microcatheter” strategy was employed to seal the potential endoleak. In brief, besides the delivering system described above, a 5F guiding catheter (Boston Scientific Co., USA) was delivered from the left femoral artery to the ipsilateral ICA, and through which an Echelon-10 (EV3, Irvine, CA, USA) microcatheter was delivered into the cavernous sinus via the fistula. Microcoils or Onyx would be delivered through the pre-set microcatheter as salvage for the unexpected endoleak after stent implantation.

Dual antiplatelet medication (aspirin 100 mg/day, clopidogrel 75 mg/day) was maintained for at least 3 months after the procedure, and aspirin alone was maintained for another 3 months.

### Clinical and graphical follow-up regime

Clinical follow-up was arranged for all patients at 1, 6, and 24 months after discharge. DSA follow-up was arranged 6 months after discharge for all patients except for the two cases who received parent artery occlusion.

## Results

### Patients’ information

Three females and 7 males were enrolled in this study. The average age was 28 ± 2.556 years (18–45 years), and the average medical course was 33.6 ± 17.39 days (2 days–6 months). Eight patients were treated for the first time, and the other 2 had been treated previously elsewhere. The typical symptoms include chemosis, exophthalmos, intracranial bruit, and ocular movement disorder. Detailed information of these patients was summarized in Table 1.

**Table 1:** Patient information

Patient no.	Sex (F/M)	Years	Presentation	Duration	Recurrent (yes/no)	Operation process	Immediate angiographic result	Follow-up result
1	F	25	Chemosis	7 days	No	One stent	Patent, no endoleak	No symptom. Patent, no stenosis
2	M	28	Chemosis/exophthalmos/bruit/ocular movement disorder	5 days	No	One stent, repeated dilation, persistent endoleak, second stent, endoleak, occlude the ICA with balloons	ICA occlusion	Decreased visual acuity. No DSA follow-up
3	M	45	Chemosis	12 days	No	Could not deliver the stent to the fistula, occlude the ICA with balloons	ICA occlusion	No symptom. No DSA follow-up
4	M	21	Chemosis/exophthalmos/bruit/ocular movement disorder	6 days	No	One stent, endoleak, seal the fistula with coils	Patent, no endoleak	Slight ocular movement disorder. Patent, DSA follow-up in a local hospital.
5	M	19	Chemosis/exophthalmos/bruit	14 days	No	One stent	Patent, no endoleak	No symptom. Patent, no stenosis
6	F	29	Chemosis/exophthalmos	3 days	No	One stent	Patent, no endoleak	No symptom. Patent, no stenosis
7	F	18	Chemosis/exophthalmos/bruit/ocular movement disorder	4 days	No	One stent	Patent, no endoleak	No symptom. Patent, no stenosis
8	M	31	Chemosis/bruit	2 months	Yes	One stent	Patent, no endoleak	Decreased visual acuity. Refuse DSA follow-up
9	M	29	Chemosis	6 months	Yes	One stent	Patent, no endoleak	No symptom. Patent, no stenosis
10	M	35	Chemosis/exophthalmos	1.5 months	No	One stent, repeated dilation	Patent, slight endoleak	No symptom. Patent, slight stenosis, no endoleak

F, female; M, Male; ICA, internal carotid artery; DSA, digital subtraction angiography

### Immediate angiographic results

Willis covered stents were successfully delivered and released in 9 of the 10 patients, except for one case due to the tortuous ICA, for whom the parent artery was occluded with detachable balloons after an occlusion test. Immediate blockage of the abnormal shunt was achieved in 6 patients with one stent for each patient. Endoleak occurred in 3 patients. For one patient, repeated balloon inflation dramatically diminished the endoleak (patient 10). For another patient, endoleak remained even after repeated balloon dilation and another stent deployment, and this patient got parent artery occlusion with balloons after an occlusion test. The blood flow was compensated by the anterior and posterior communicating artery (patient 2). For the third patient, the endoleak was sealed with coils through a pre-set microcatheter (case 4, Fig. 4, patient 4).

### Clinical and graphical follow-up results

Symptoms gradually relieved after the operation in all patients. Clinical follow-up 1 month after discharge showed disappear of chemosis and no new neurological deficits in all patients. Ocular motor disturbance disappeared in two patients (patients 2 and 7) and was relieved in another one (patient 4). Clinical follow-up 6 and 24 months after initial discharge showed no related neurological defects in all patients except for decreased visual acuity in patient 2 and slight oculomotor paralysis in patient 4.

Angiographic follow-up was available in 7 patients 6 to 10 months after initial discharge. Slight in-stent stenosis was found in 1 case (patient 10) at the 6-month follow-up. In the patient with a slight endoleak at discharge (patient 10), the endoleak disappeared. For the two patients who received parent artery occlusion, DSA follow-up was not arranged for their silent symptoms. One patient (patient 8) refused the DSA follow-up for intact neurological function and economic burden.

### Illustrative cases

#### Case 1 (patient 1)

**Fig. 1:**
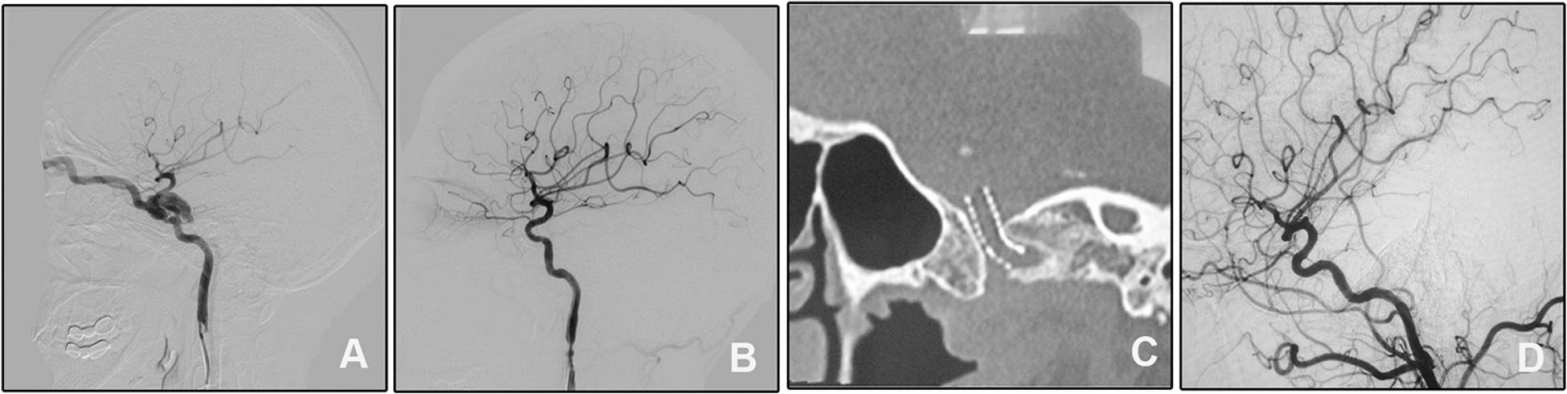
A direct CCF was cured by a Willis stent deployment. **A** DSA of the right ICA showed a direct CCF and was drained by the superior ophthalmic vein. **B** A Willis covered stent was delivered and dilated to cover the fistula and immediate angiography showed perfect repair of the vascular lumen. **C** DynaCT showed a complete inflated stent. **D** Angiographic follow-up 6 months after discharge showed patency of the ICA and no obvious in-stent stenosis

A 25-year-old girl was transferred to our department for the right chemosis for 7 days after a vehicle accident. She was diagnosed with right CCF by a six-vessel angiography. The fistula was successfully repaired with 1 Willis covered stent. At 1-month follow-up, the right chemosis disappeared with no new neurological deficits. Angiographic follow-up 6 months after discharge showed patency of the parent artery with no stenosis (Fig. 1). Clinical follow-up 6 and 24 months after initial discharge showed no neurological deficit.

#### Case 2 (patient 8)

**Fig. 2:**
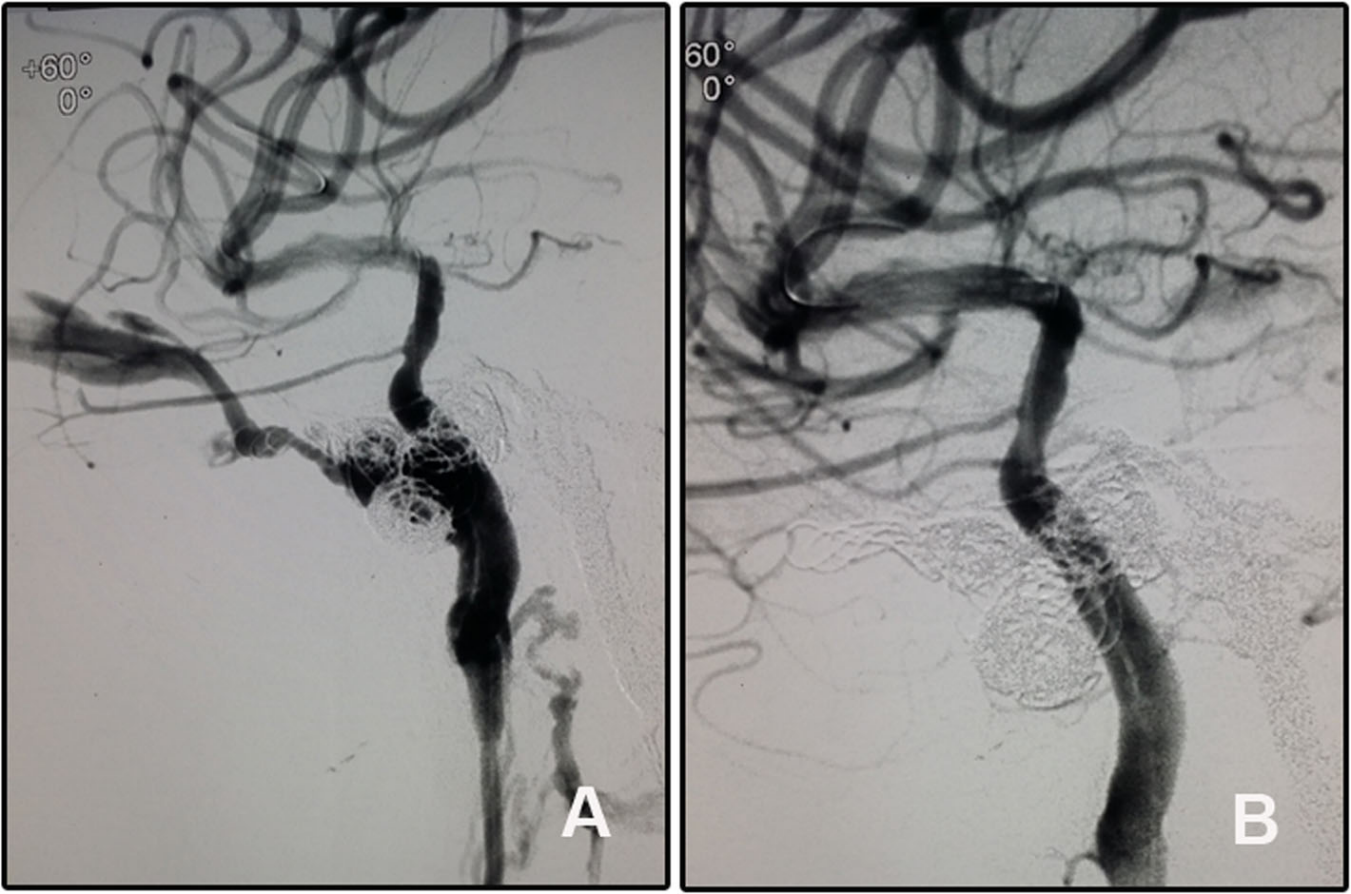
A recurrent CCF was treated with Willis covered stent. **A** The fistula was located at the cavernous segment of the right ICA and was drained by the superior ophthalmic vein. The embolization materials of microcoils and Onyx were seen. **B** Angiography after the stent deployment showed disappear of the fistula and patency of the parent artery

A 31-year-old man was admitted for right chemosis and intracranial bruit for 2 months. He had head trauma 2 months ago and was diagnosed with traumatic CCF. He received embolization with coils and Onyx-18 in a local hospital. The signs were relieved just after the operation but aggravated in the last 10 days before being transferred to our hospital. DSA confirmed recurrence of the CCF, and the fistula was repaired by a Willis covered stent. The bruit disappeared just immediately after the operation and the chemosis gradually relieved and disappeared at one-month follow-up after discharge (Fig. 2). This patient refused DSA follow-up as the economic burden. Clinical follow-up at 6 and 24 months showed disappearance of the signs but decreased right visual acuity.

#### Case 3 (patient 10)

**Fig. 3:**
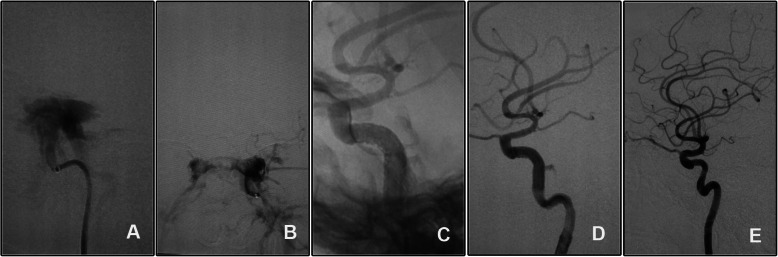
The endoleak after Willis covered stent deployment disappeared spontaneously. **A**, **B** Angiography of the left ICA showed a high flow CCF of the cavernous segment. **C**, **D** The endoleak persisted although repeated balloon inflation. **E** Follow-up angiography showed disappear of the endoleak and a slight in-stent stenosis

A 35-year-old man, who suffered from severe head trauma and subsequent intracranial hematoma evacuation and decompressive craniectomy 1.5 months ago, came to our department for persistent left chemosis and exophthalmos. DSA confirmed a left high flow CCF. A slight endoleak was encountered even after repeated balloon inflation and second stent deployment. His eye signs gradually diminished and completely disappeared at the one-month clinical follow-up. Angiographic follow-up showed disappear of the endoleak and slight in-stent stenosis (Fig. 3). Clinical follow-up 6 and 24 months showed no neurological signs.

#### Case 4 (patient 4)

**Fig. 4:**
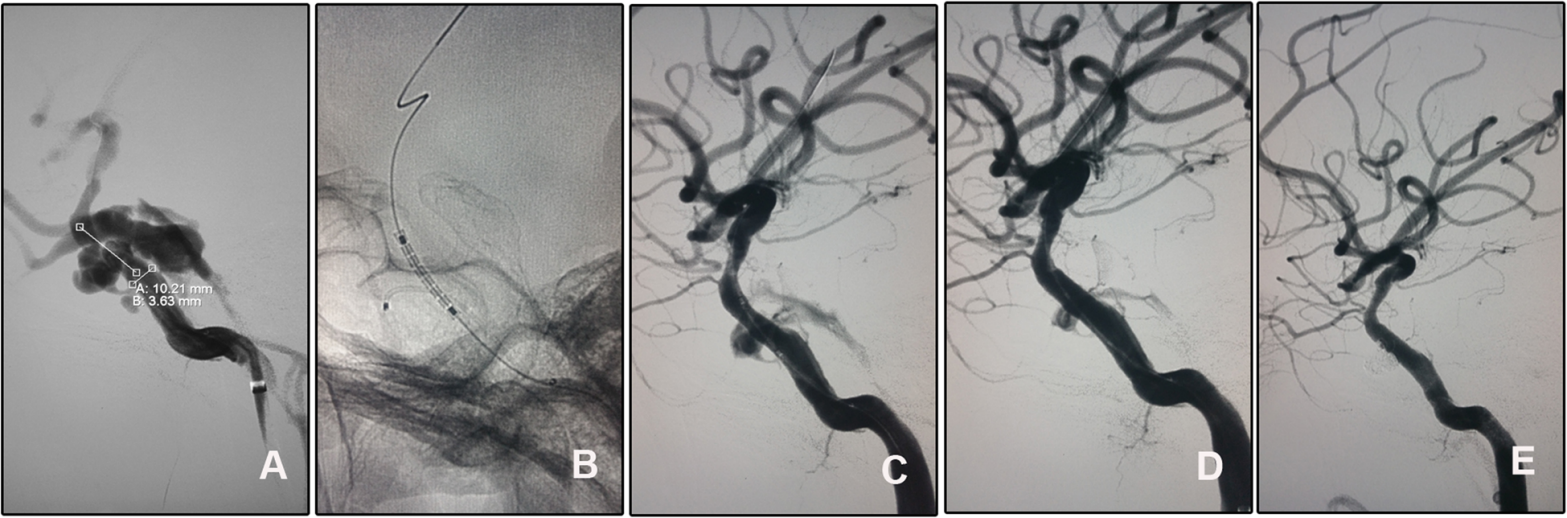
The endoleak after stent deployment was sealed with coils through a pre-set microcatheter. **A** Angiography of the right ICA confirmed the diagnosis and showed the fistula. **B** A microcatheter was placed into the cavernous sinus through the fistula. **C**, **D** After the stent deployment and repeated balloon dilation, an endoleak persisted. **E** The endoleak was sealed with two coils delivered through the pre-set microcatheter

A 21-year-old man suffered from chemosis, exophthalmos, intracranial bruit, and ocular movement disorder for 6 days after a vehicle accident. Diagnostic angiography confirmed right CCF. To eliminate the potential endoleak, a microcatheter was pre-set into the cavernous sinus through the fistula. After stent deployment and a second balloon dilation, the unexpected endoleak was sealed with 2 coils just near the fistula (Fig. 4). His signs gradually relieved and completely resolved at 1-month follow-up. He received angiographic follow-up in a local hospital but lost the imaging data. The copied in-hospital record described the disappearance of the fistula with no in-stent stenosis. Clinical follow-up 24 months after initial discharge was normal.

## Discussion

Originally approved as a “bailout” in the event of coronary artery rupture, covered stents could reconstruct the vessel defects immediately while preserving the parent artery. Willis is the first covered stent exclusively designed for intracranial vasculature. In this paper, we shared our preliminary experience with this device for treating direct CCFs.

The goal of CCF treatment is to completely occlude the fistula while preserving the normal blood flow through the ICA. Traditionally, the treatment options include conservative treatment, surgical, and endovascular intervention [13]. Conservative management, consisting of external manual compression of the ipsilateral cervical carotid artery several times a day for 4–6 weeks, and CCF occlusion only occurred in only 17% of patients at 1-year follow-up [14]. Surgical intervention may involve suturing, clipping, or trapping the fistula, packing the cavernous sinus to occlude the fistula, sealing the fistula with fascia and glue, ligating the ICA, or a combination of these procedures. Overall success rates using surgical intervention in the treatment of CCFs have been reported at between 31 and 79% [13]. The high morbidity and mortality rates render surgical intervention only be warranted for patients in which endovascular intervention is not possible or unsuccessful. Nowadays, endovascular intervention has been the first-line therapy either via the trans-arterial or transvenous routes. Metallic coils, balloons, liquid agents, and more recently flow diverters are always used for occluding the cavernous sinus and fistula [15–18]. More than 80% of patients who undergo endovascular treatment for direct and indirect CCFs will experience a complete cure [13]. Compared with treatment modalities for CCFs with coils, Onyx, and balloons, the covered stent has some advantages: high overall complete occlusion rate, a relatively simple and rapid procedure, no coil herniation into the parent artery, no mass effect, and no CCF recanalization and recurrence. Of these advantages, the high overall complete occlusion rate is the most important [15]. However, there are also some limitations for covered stents in repairing vascular defects. First, small perforating vessel sacrifice restricts its usage. The absence of important branches at the cavernous ICA provides the feasibility of covered stents for direct CCFs. Second, the tortuous ICA makes the delivery of the stiff stent difficult. The Willis covered stent is designed exclusively for intracranial vasculature, aiming to overcome the inherent disadvantages of traditional covered stents [19]. Firstly, the abundance in versions with various diameters and lengths can accommodate target vessels of various sizes. Second, the unique structure design makes it more flexible for delivery. Third, the low inflation pressure of the balloon for releasing the stent decreases the potential of target vascular damage. Finally, the trackability of this stent has greatly improved than its congeners. A strong supporting system is necessary for delivering stiff covered stents [20]. We proposed an effective delivery system as described above, which was consisted of 8F guiding catheter, 5F Navien catheter, XT-27 microcatheter, and an exchanging microwire in co-axis. In our series, all stent deliveries with this system were successfully. In patient 3, unavailability of the soft Navien catheter lead to stent delivery failure. These results proved the necessity and efficacy of our delivering system.

From the economical perspective, detachable balloons are much cheaper than coils combined with liquid embolism in china with comparable complete occlusion rate and recanalization rate [21]. Others reported a higher recanalization rate of detachable balloons [22]. In our initial experience, the cost of using Willis covered stent for this disease is similar to that of coils combined liquid embolism. However, Willis covered stent harbor the advantage of no cavernous packing and resultant potential of cranial nerve injury. More cases are needed to further elucidate the economic issue for this novel device.

Defined as persistent perfusion of the space between the stent graft and parent vessel wall [23], endoleak occurred in 38.89% cases immediately after covered stent deployment [24], which was consistent with our cases (30%, 3/10). As major endoleak remained even after repeated balloon dilation and second stent deployment, we had to sacrifice the ICA with balloons in patient 2. In patient 10 (case 3, Fig. 3), endoleak diminished after re-dilation of the balloon and disappeared spontaneously at 6-month follow-up. In patient 4 (case 4), the endoleak was sealed with two coils via a pre-set microcatheter (Fig. 4). As we knew, this is the first report to pre-set a microcatheter for unexpected endoleak after stent deployment. As a pre-set microcatheter may induce a gap between the arterial wall and the stent leading to persistent endoleak, it is more reasonable to deliver the microcatheter via the venous route. Oblate vessel wall, diameter variance of the covered vessel, sharp bone fracture that stabbing the stent membrane, vasospasm, and stent diameter mismatch may contribute to immediate endoleak [24]. Balloon re-dilation and additional stent deployment were reported to solve this complication, and slight endoleak might disappear spontaneously in the follow-up [23]. The efficacy of these strategies was validated in patient 10 (case 3, Fig. 3). In this patient, the endoleak dramatically relieved after repeated balloon dilation, and the residual endoleak disappeared spontaneously at the 6-month angiographic follow-up. For patient 2, the parent artery was sacrificed as persistent endoleak despite repeated balloon dilation and second stent deployment.

In-stent stenosis was reported to occur in 18.0% and 20.9% of patients after Willis stent implantation at 2 and 6 years follow-up [24]. In our series, in-stent stenosis was seen in 1 case at 6-month follow-up (10.0%, case 3, Fig. 3). Smoking and stent angulation were predictors of late in-stent stenosis [24]. Repeated balloon dilation induced intima damage may contribute to the aggressive intima hyperplasia in this patient. Aspirin was prescribed and intermittent clinical follow-up was scheduled for him.

Recurrent or partially occluded CCF is always a nightmare for physicians, as the delivery of the microcatheter may be extremely difficult. Under this condition, a covered stent may serve as an alternative. In patient 8 (Fig. 2, case 2), the inferior petrosal sinus had already been occluded in the initial operation and a covered stent was selected as the salvage treatment. The fistula was occluded immediately after stent deployment.

## Limitations

There are some limitations to this work. First, only 10 patients were enrolled in the study. The small series could only provide preliminary experience for this novel device for CCFs, a conclusive statement could not be induced from this study. More cases should be enrolled to further elucidate the efficacy of this technique for CCFs. Second, the small series make statistical analysis of the results and comparison with other treatment modality impractical. Third, patient enrollment criteria in this study were mainly subjectively determined by a group of senior neuro-interventionalists, and the patients and their relatives finally determined the treatment modality. This makes this series a biased study. Forth, the small series failed to analyze the risk factors for operation-related complications such as endoleak.

## Conclusions

Despite limitations, our initial results indicated that Willis covered stent is feasible and can be considered as an alternative in treating direct CCFs. More cases are needed to further elucidate this issue.

## Data Availability

The datasets used during the current study are available from the corresponding author on reasonable request.

## Abbreviations

CCF: Carotid cavernous fistula
DSA: Digital subtraction angiography
ICA: Internal carotid artery
